# Publisher Correction: Disease-relevant transcriptional signatures identified in individual smooth muscle cells from healthy mouse vessels

**DOI:** 10.1038/s41467-018-07887-3

**Published:** 2018-12-17

**Authors:** Lina Dobnikar, Annabel L. Taylor, Joel Chappell, Phoebe Oldach, Jennifer L. Harman, Erin Oerton, Elaine Dzierzak, Martin R. Bennett, Mikhail Spivakov, Helle F. Jørgensen

**Affiliations:** 1 Nuclear Dynamics Programme, Babraham Institute, Babraham Research Campus, Cambridge CB22 3AT, UK; 2 Division of Cardiovascular Medicine, University of Cambridge, Cambridge Biomedical Campus, Cambridge CB2 0QQ, UK; 3 MRC Centre for Inflammation Research, University of Edinburgh, Little France Crescent, Edinburgh EH16 4TJ, UK; 4 Functional Gene Control Group, Epigenetics Section, MRC London Institute of Medical Sciences, Du Cane Road, London W12 0NN, UK; 5 Institute of Clinical Sciences, Faculty of Medicine, Imperial College, Du Cane Road, London W12 0NN, UK; 6 Sir William Dunn School of Pathology, University of Oxford, South Parks Rd, Oxford OX1 3RE, UK; 7 Present Address: Centre for Molecular Informatics, Department of Chemistry, University of Cambridge, Lensfield Road, Cambridge CB2 1EW, UK

Correction to: *Nature Communications*; 10.1038/s41467-018-06891-x, published online 01 November 2018

The original version of this Article contained errors in the author affiliations.

Martin R. Bennett was incorrectly associated with Nuclear Dynamics Programme, Babraham Institute, Babraham Research Campus, Cambridge, CB22 3AT, UK. This has now been corrected in both the PDF and HTML versions of the Article.

Furthermore, Phoebe Oldach was incorrectly associated with Centre for Molecular Informatics, Department of Chemistry, University of Cambridge, Lensfield Road, Cambridge, CB2 1EW, UK.

This has now been corrected in the HTML version of the Article. The PDF version of the Article was correct at the time of publication

