# Supplementary material for: Disease-relevant transcriptional signatures identified in individual smooth muscle cells from healthy mouse vessels
Source: Nat Commun. 2018 Nov 1;9:4567. doi: 10.1038/s41467-018-06891-x (PMC6212435; doi:10.1038/s41467-018-06891-x)
Supplement: Supplementary file 1 — Supplementary Information [file 41467_2018_6891_MOESM1_ESM.pdf]

Supplementary Information

Disease-relevant transcriptional signatures identified in individual smooth muscle cells from healthy mouse vessels

Dobnikar et al

Supplementary Figures 1-11
Supplementary Table 1
Supplementary Data files 1-9 (Excel files)

Supplementary Figure 1

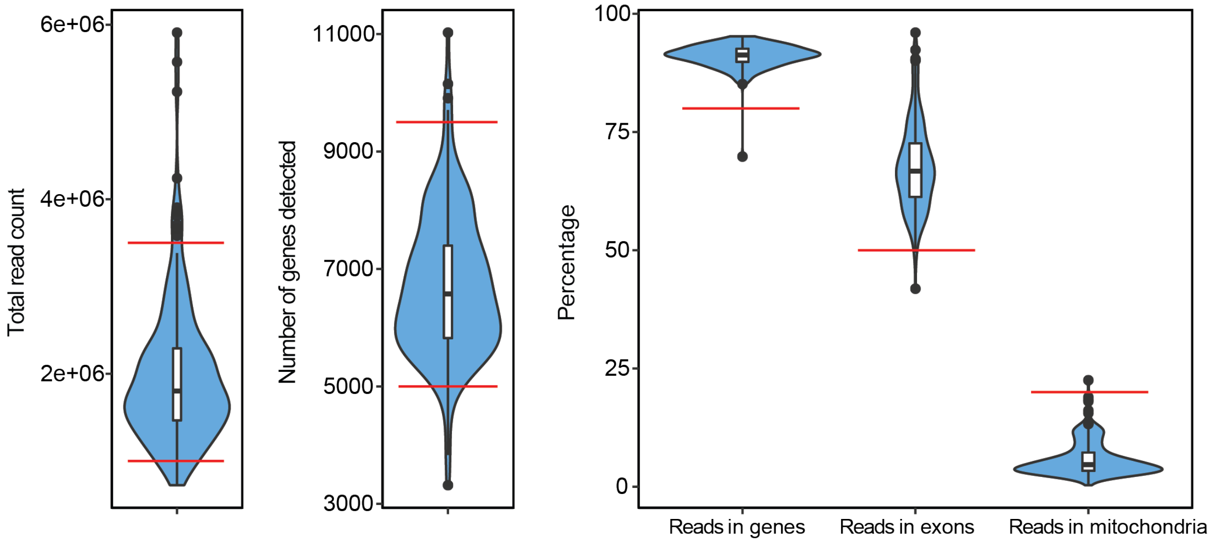

Supplementary Figure 1. Quality control of the profiled *ex vivo* single VSMC transcriptomes.
Violin plots showing the quality control metrics of total read count, number of genes detected, percentage of reads in genes, percentage of reads in exons and percentage of reads in mitochondria per cell. Red lines indicate the thresholds used for filtering out poor-quality cells.

Supplementary Figure 2

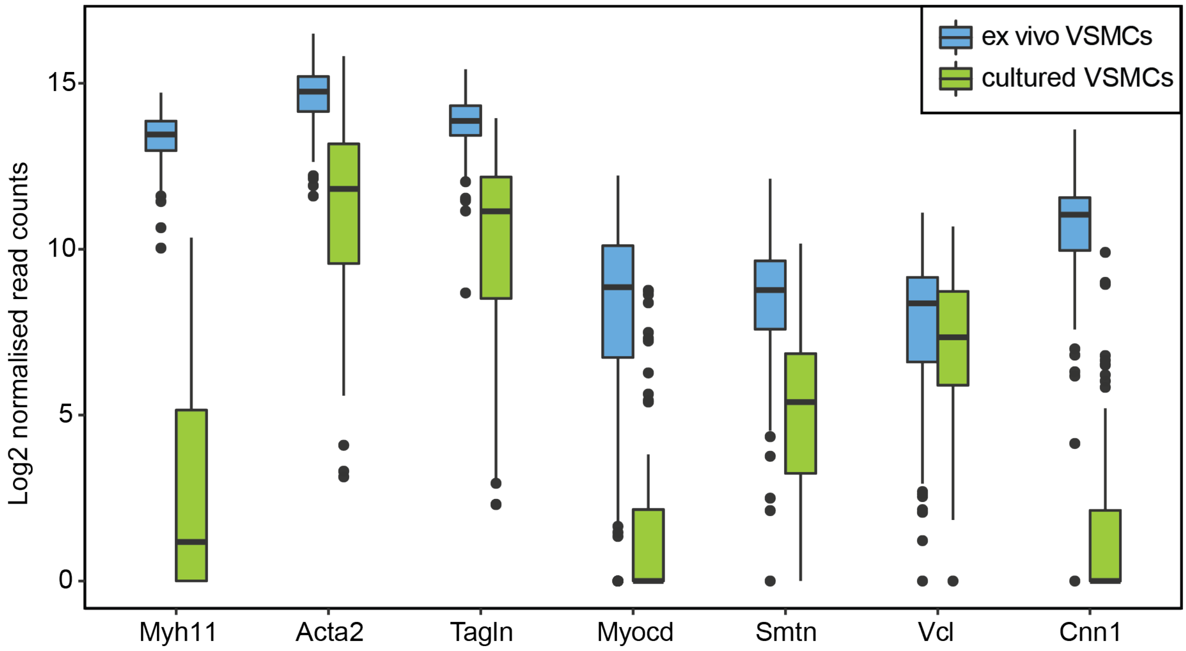

Supplementary Figure 2. Cultured VSMCs show reduced expression of VSMC marker genes compared with *ex vivo* VSMCs.

Boxplot showing normalised $\log_2$-transformed read counts for *Myh11*, *Acta2*, *Tagln*, *Myocd*, *Smtn*, *Vcl* and *Cnn1* genes for *ex vivo* (blue) and cultured (green) VSMCs. Median (centre line), first and third quartiles (bounds of box), 1.5 interquartile range (whiskers) and outliers (dots) are shown. Data for cultured VSMCs were obtained from GEO Accession GSE79436.

Supplementary Figure 3

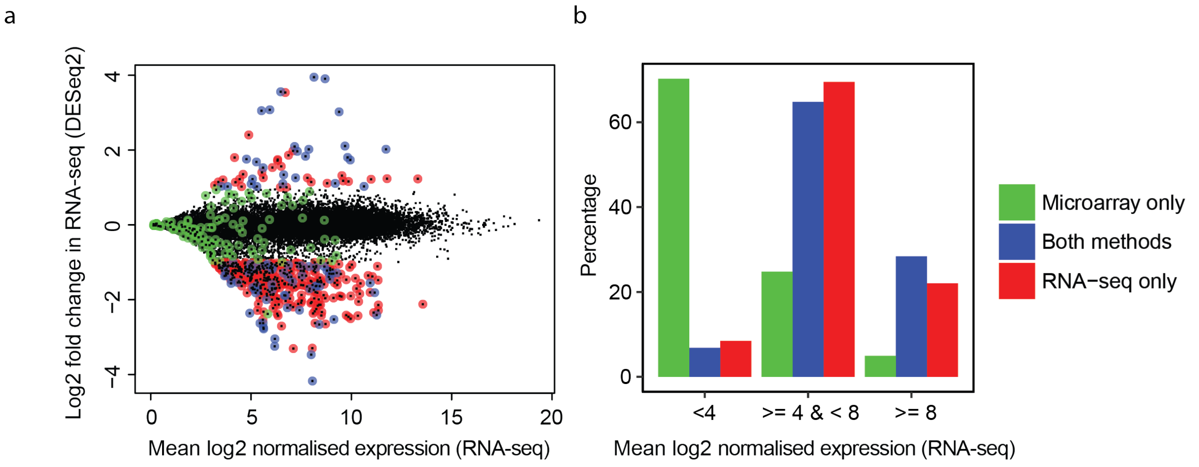

Supplementary Figure 3. Comparison of microarray and RNA-seq methods for identifying differentially expressed genes between the AA and DT regions.

(a) Scatterplot showing log_2-normalised mean expression and log_2-fold change in expression between the AA and DT regions for all genes detected using bulk RNA-seq (black dots). Genes identified as differentially expressed using RNA-seq method only (red), microarray method only (green) and both methods (blue) are shown.

(b) Bar chart showing the percentages of differentially expressed genes detected by microarray only (green bars), RNA-seq (red) or both methods (blue) for different expression level thresholds.

Supplementary Figure 4

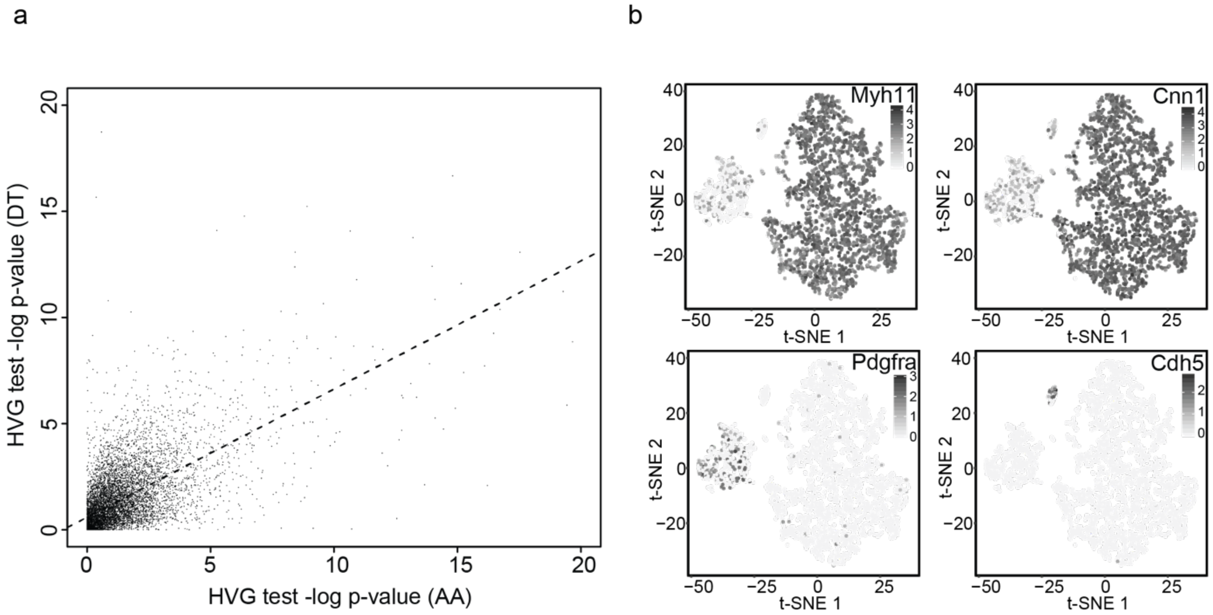

Supplementary Figure 4. Correlation of the Highly Variable Gene scores for AA and DT regions.
(a) Relationship between HVG test –log-p-values detected for each gene across AA and DT cells, respectively.
(b) Expression of VSMC (*Myh11/Cnn1*), adventitial (*Pdgfra*) and endothelial (*Cdh5*) markers used to identify these populations in the t-SNE clustering of the 10X Chromium dataset from the whole aorta shown in Figures 4d-e.

Supplementary Figure 5

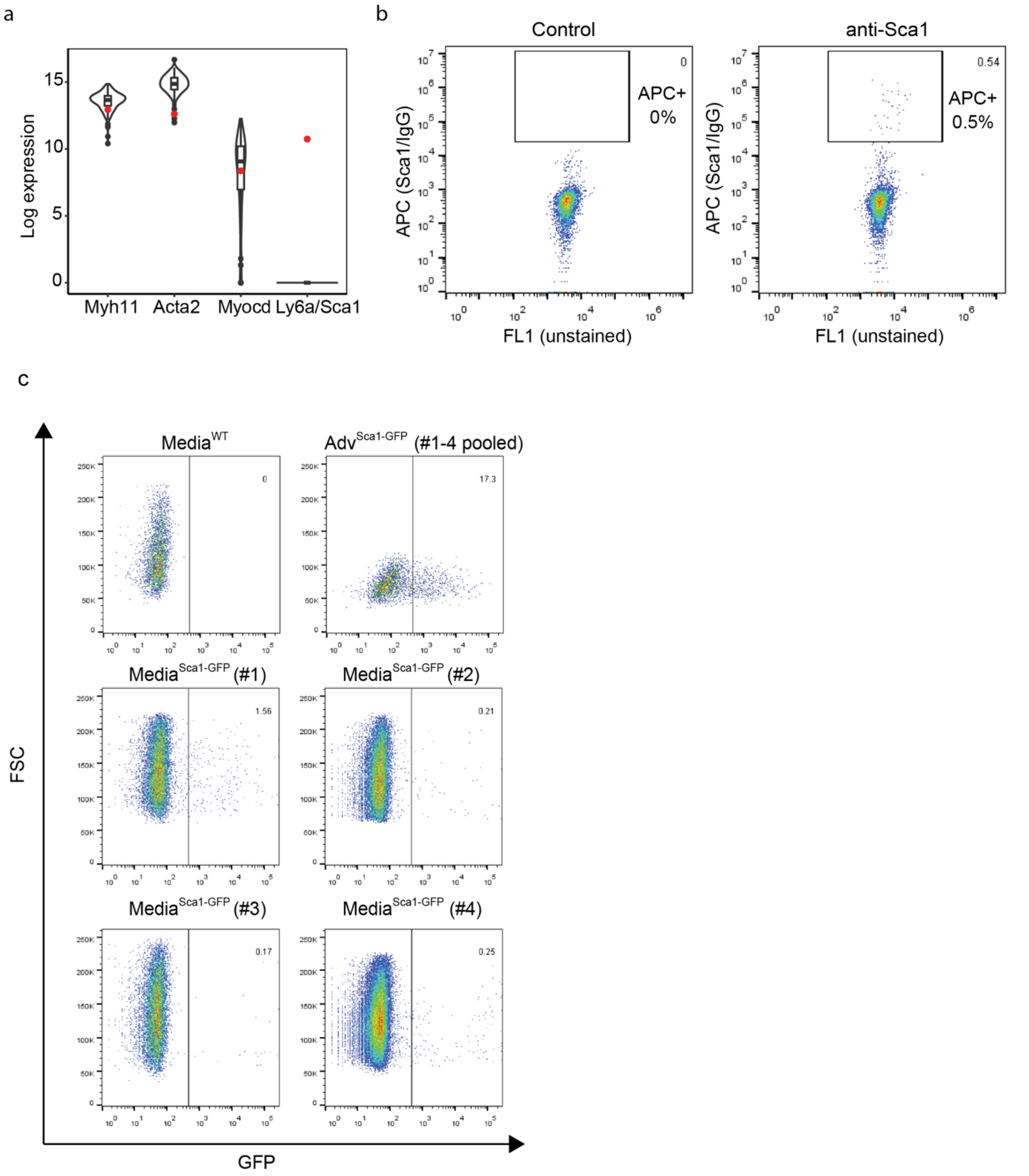

Supplementary Figure 5. A small number of medial cells express Sca1.
(a) Violin plots showing expression of *Myh11*, *Acta2*, *Myocd* and *Ly6a/Sca1* transcripts in cells analysed with Fluidigm C1, with the cell expressing *Ly6a/Sca1* marked in red.
(b) FACS plots of APC detection in medial cells from wild type animals stained with APC-coupled IgG control (left panel) or anti-Sca1 antibody (right panel) against FL1 intensity.
(c) FACS plots of GFP expression (with forward scatter, FSC, on y-axis) in medial cells isolated from wild type (WT) medial controls (n=1), and adventitial (Adv, n=1) and medial cells (n=4) isolated from Sca1-GFP animals. The percentage of GFP-positive cells is shown.

Supplementary Figure 6

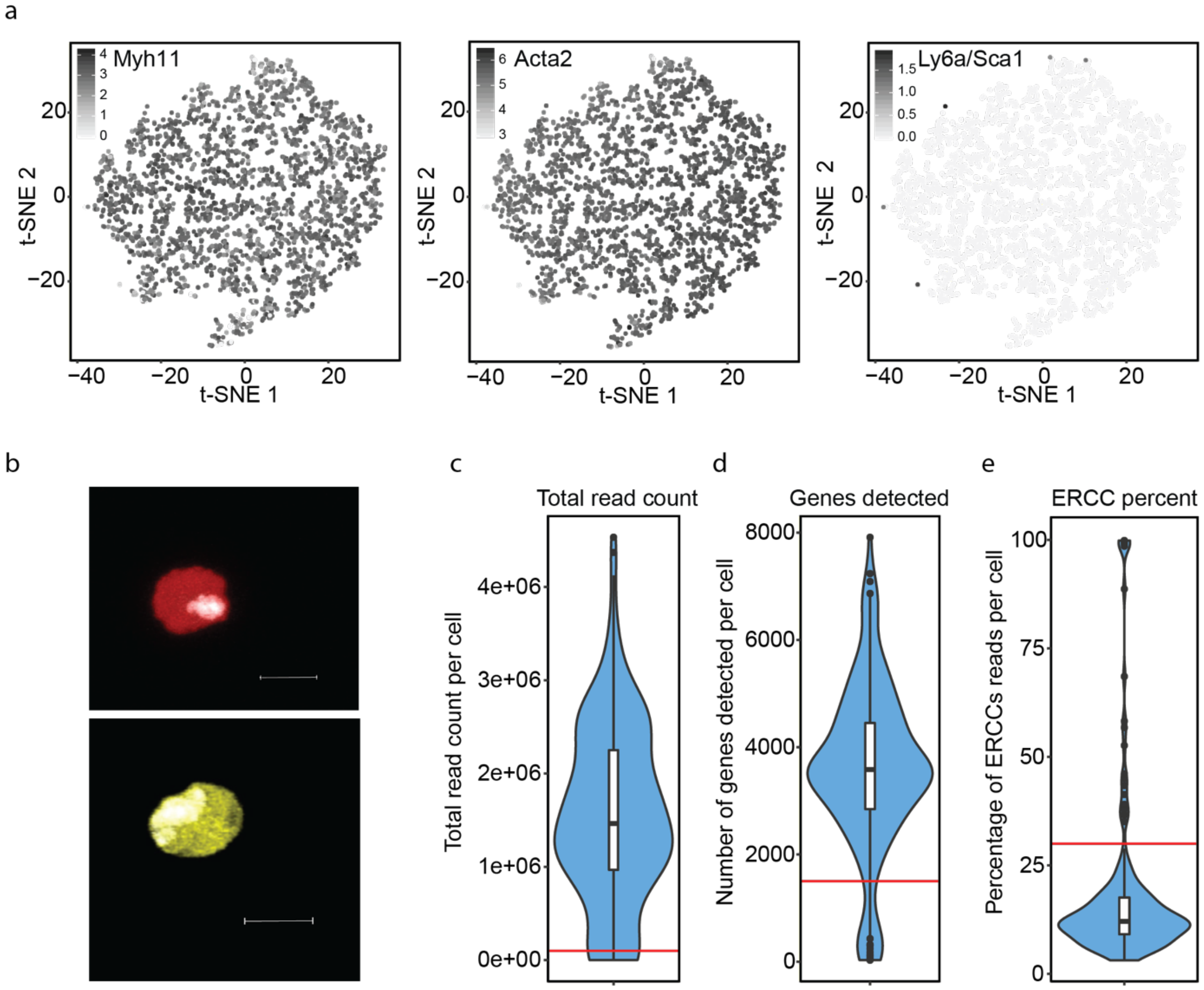

Supplementary Figure 6. A small number of VSMC-lineage cells express Sca1.

(a) t-SNE plots showing 10X Chromium profiling of 2840 VSMC lineage-positive cells isolated from a pool of the medial layer from three Myh11-CreERt2/Confetti animals, with expression of *Myh11*, *Acta2* and *Ly6a/Sca1* colour-coded from white to dark grey.

(b) Confocal images of two representative medial cells obtained by FACS sorting for one of the four fluorescent reporter proteins from the Confetti system, demonstrating that single cells are isolated following this strategy. Red: RFP, Yellow: YFP, white: DAPI. Scale bars are 10 μm.

(c-e) Violin plots showing the quality control metrics of total read count (c), number of genes detected (d) and the percentage of ERCC reads per cell (e) in single cells profiled using the Smart-seq2 protocol. Red lines indicate the thresholds used for filtering out poor quality cells.

Supplementary Figure 7

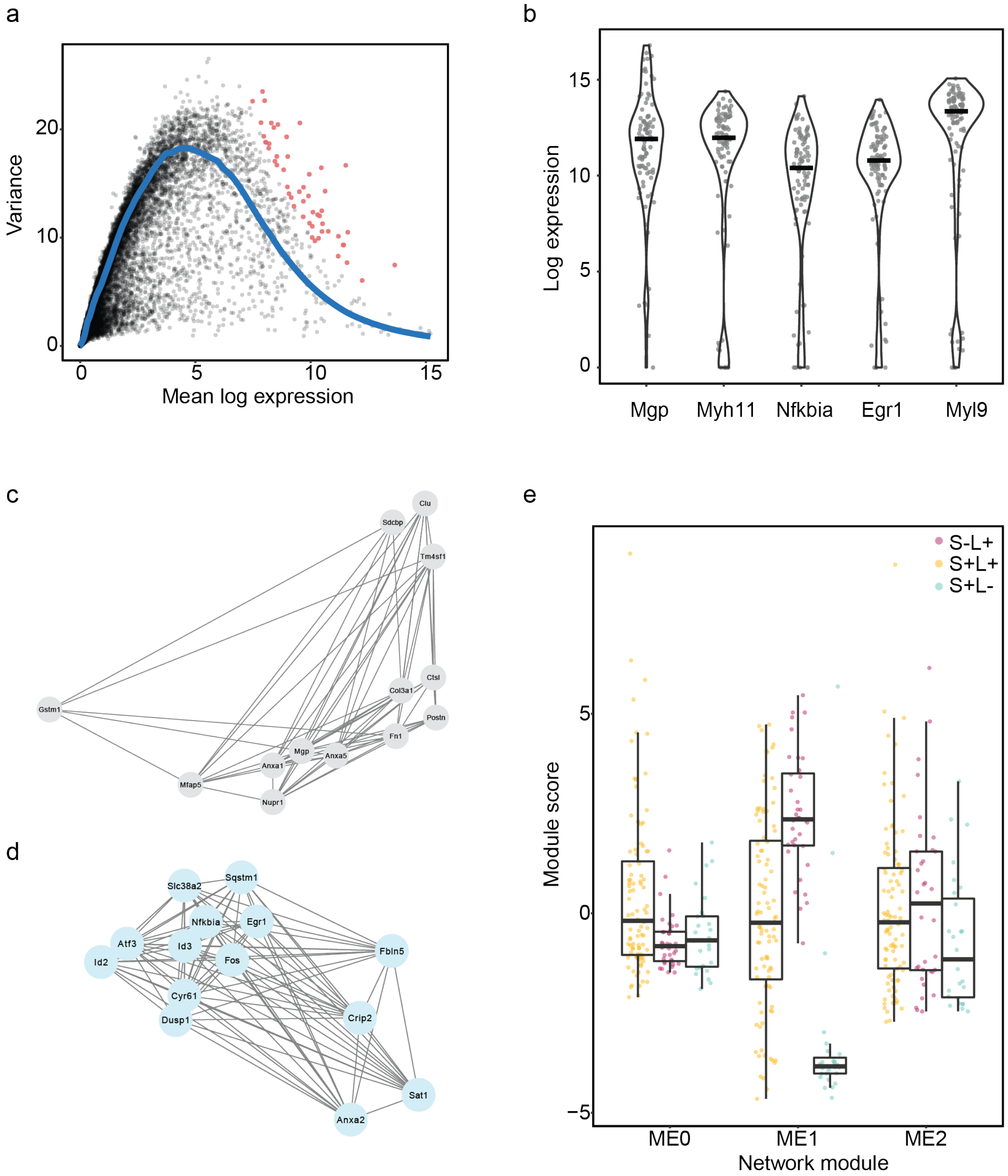

Supplementary Figure 7. Highly variable genes (HVGs) detected in S+L+ cells.
(a) Scatter plot showing the mean-variance relationship of log_2-transformed expression levels for each expressed gene in the S+L+ population. Genes detected as HVGs (adjusted p-value < 0.05) are highlighted in red.
(b) Dot plots showing log-transformed normalised counts detected in individual S+L+ cells for selected genes detected as HVGs across single cells.
(c, d) Network graphs showing co-expressed modules of genes ME0 (c) and ME2 (d) identified by WGCNA (ME1 module is shown in Figure 7d).
(e) Boxplots showing summarised expression scores (Module scores) of ME0, ME1 and ME2 module genes in S-L+ (magenta), S+L+ (yellow) and S+L- (blue) cells. Median (centre line), first and third quartiles (bounds of box), 1.5 interquartile range (whiskers) and scores for each cell (dots) are shown.

Supplementary Figure 8

a

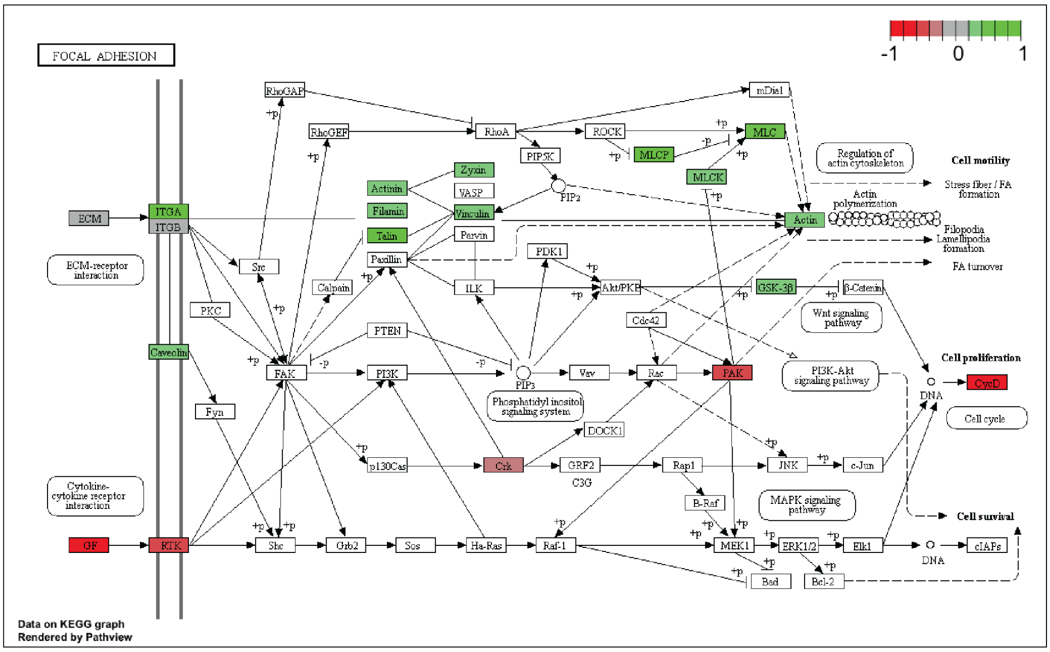

b

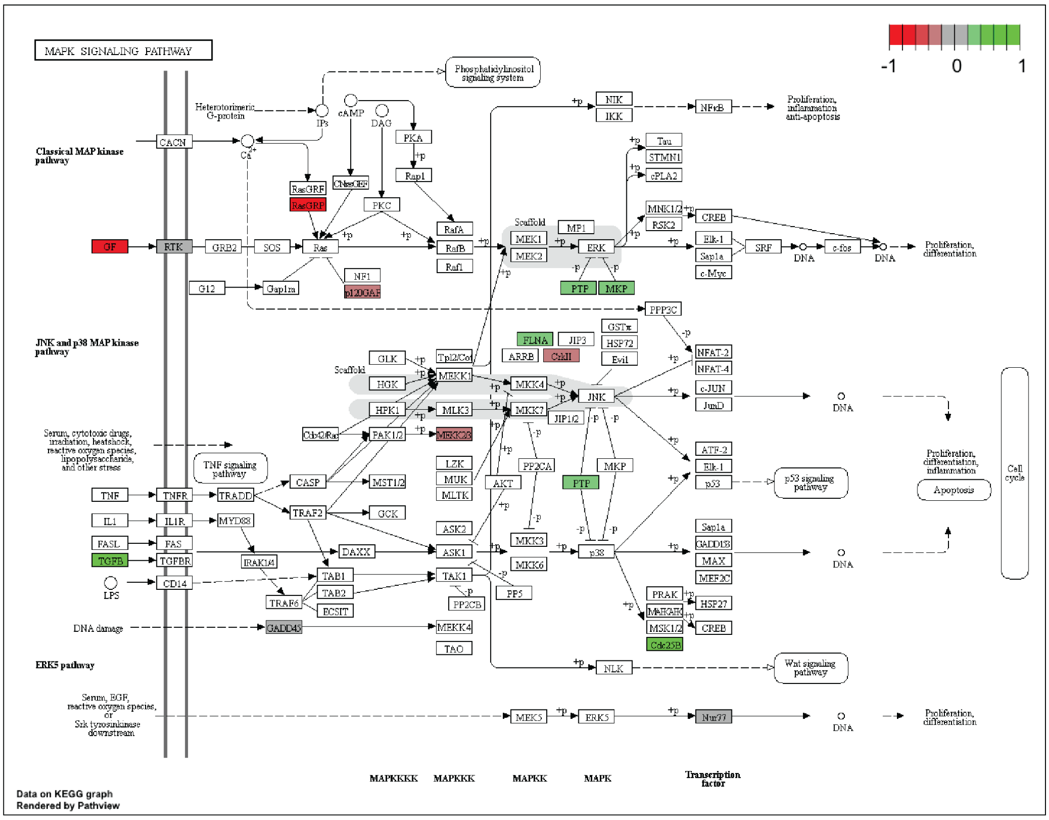

Supplementary Figure 8. Significant correlation with cVSMC score for genes in selected KEGG pathways. (a, b) KEGG pathways for focal adhesion (a) and MAPK signalling (b) with positive (cVSMCpos, shades of green) or negative (cVSMCneg, shades of red) correlations of expression levels with the cVSMC scores shown for significantly correlated genes.

Supplementary Figure 9

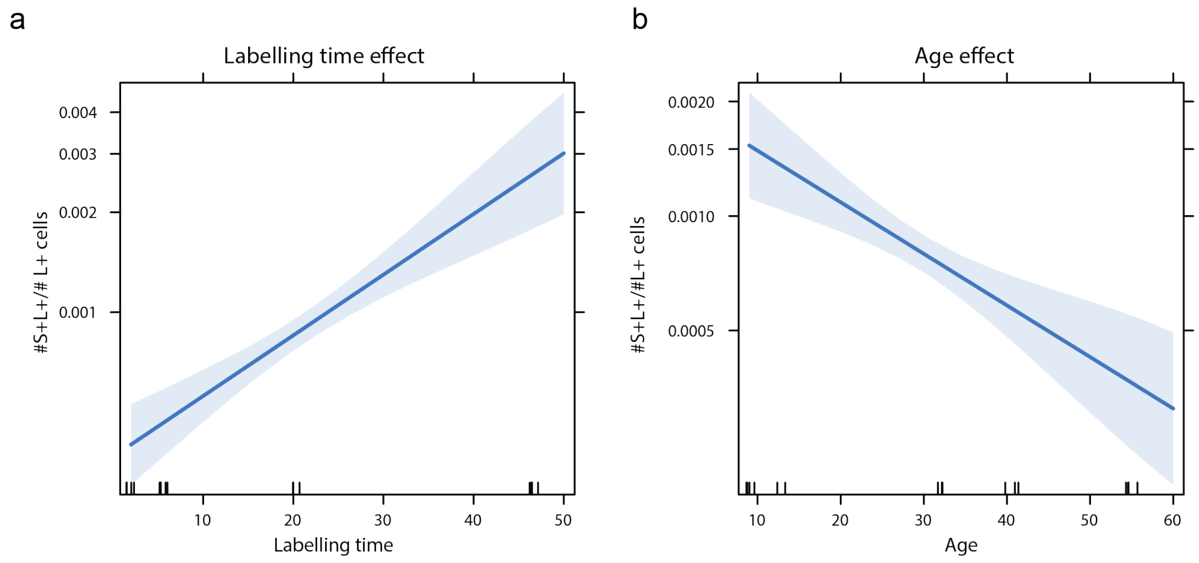

Supplementary Figure 9. Bivariate analysis of the dependence of the proportion of S+L+ cells on labelling time and animal age *in vivo*.

Effect plots showing the results of a bivariate logistic regression using the proportion of S+L+ cells among L+ cells (#S+L+/#L+ cells) as the response variable, and labelling time and age as explanatory variables. (a) Effect of labelling time: logit-link regression coefficient = 0.042+/-0.014, p-value = 2.72e-09 based on Student's distribution; (b) effect of age: logit-link logistic regression coefficient = -0.028+/-0.015, p-value = 3.07e-5 based on Student's distribution. Regression trendlines and 95% confidence intervals (shaded areas) are plotted.

Supplementary Figure 10

a

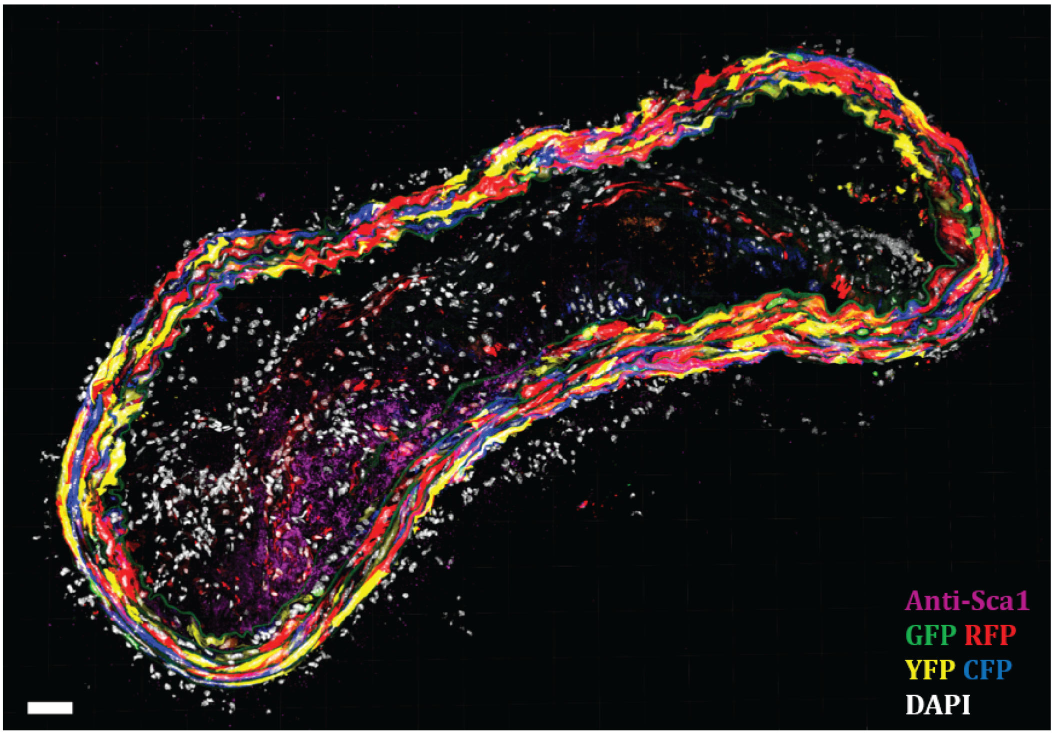

b

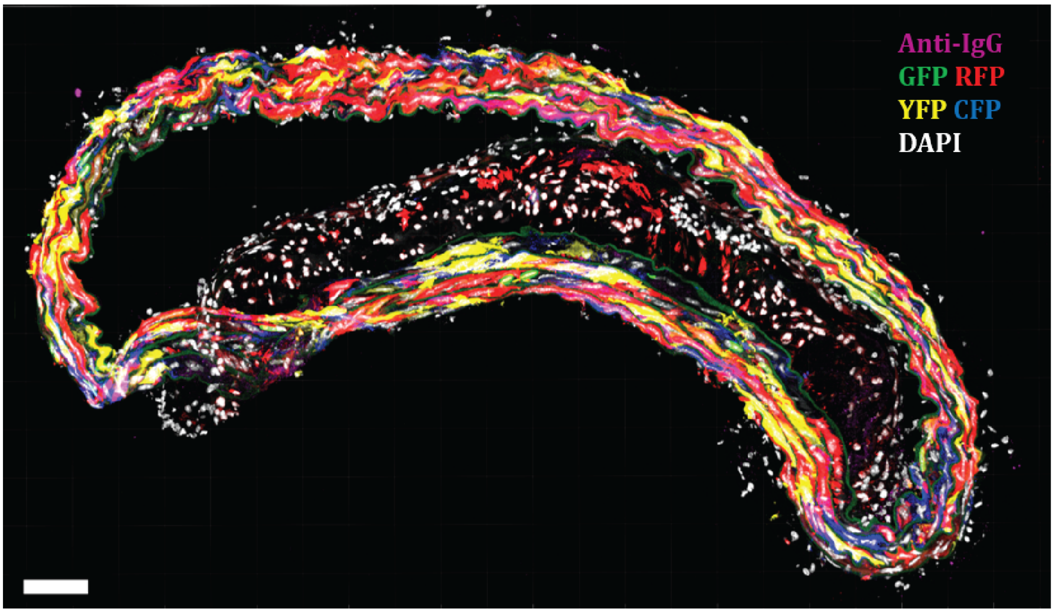

Supplementary Figure 10. Sca1 expression in aortic plaques induced by a cholesterol-rich diet. Serial cryosections of the descending thoracic aorta from an Myh11-CreERt2/Confetti/ApoE$^{-/-}$ mouse fed a cholesterol-rich diet for 30 weeks, stained with APC-coupled anti-Sca1 (a) or IgG control (b) antibody. Signals for fluorescent proteins (GFP, green; RFP, red; YFP, yellow and CFP, blue), nuclear DAPI (white) and APC-coupled antibody (magenta) are shown in each panel. Images show maximum projections of 16 (a) or 23 (b) 2 μm Z-slices (2 μm each). Scale bars are 20 μm (a) or 15 μm (b).

Supplementary Figure 11

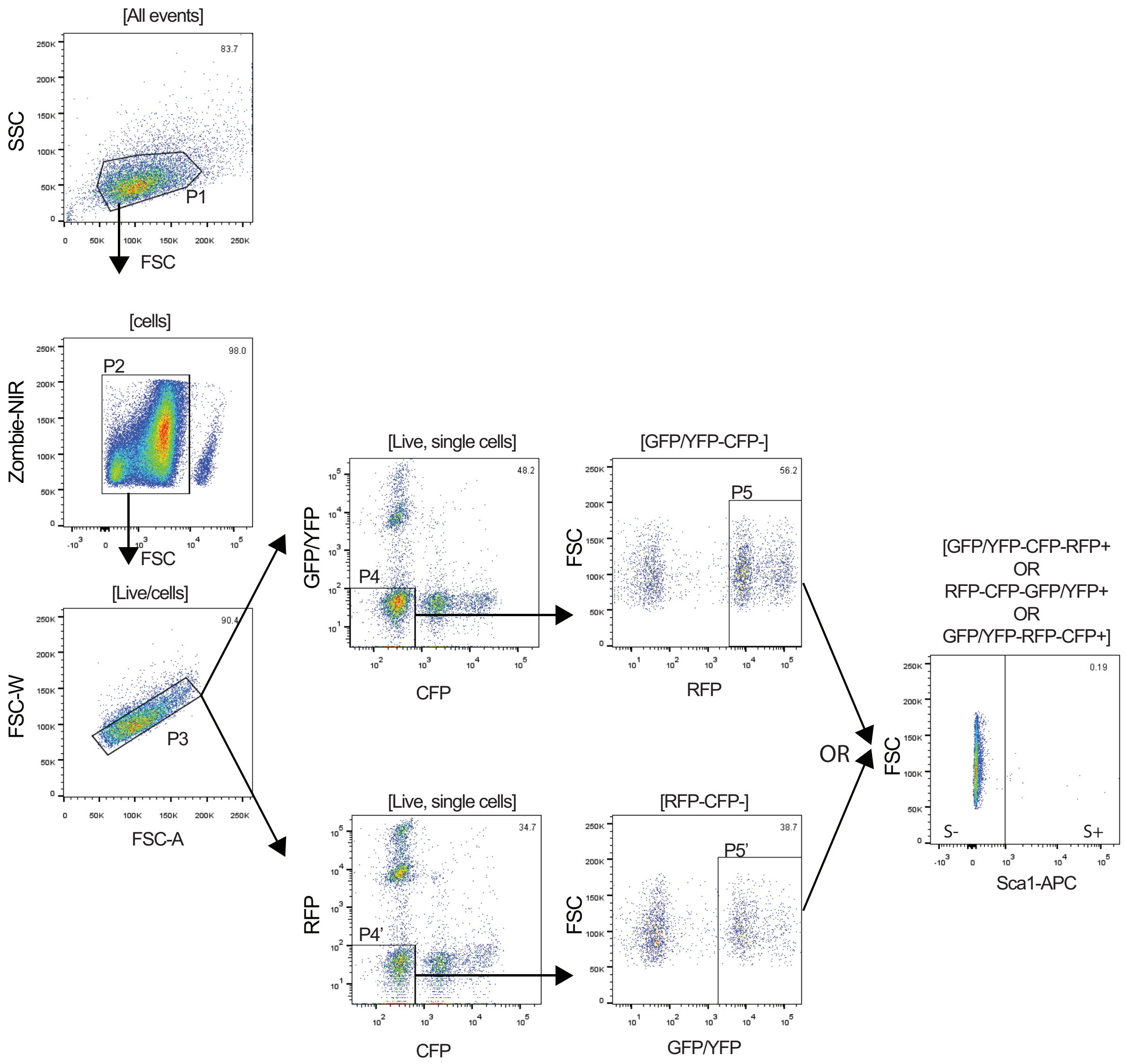

Figure 11. Example flow cytometry gating strategy used to sort lineage+ Sca1+ cells.
To isolate Sca1-positive, Lineage positive (S+L+), Sca1-positive, Lineage negative (S+L-) and Sca1-negative, Lineage positive (S-L+) cells from tamoxifen labelled Myh11-CreERt2/Confetti animals, events were first gated for cells (forward scatter (FSC)/side scatter (SSC), P1), live (Zombie-near infrared (NIR) negative, P2; staining for live cells was done for a subset of the samples, as described in the Methods section) and singlets (FSC-A/FSC-W, P3). Gates for Zombie-NIR were set based on an unstained cell sample. Events gated for live, single cells were then used to select cells expressing only a single lineage tracing fluorescent protein label to further eliminate doublets. Live, single VSMCs were gated negative for two Confetti markers (P4, P4') and this double negative population used to select VSMCs that are positive for the third Confetti marker (P5, P5'). VSMCs from wild type animals were used to define the gates for Confetti markers. VSMCs positive for only a single Confetti marker were combined using a Boolean "OR" gate and subsequently gated for Sca1 expression by anti-Sca1-APC staining (S+ or S-). Gates for Sca1-selection was defined using anti-Sca1-APC stained adventitial cells (positive) and VSMC samples stained with an APC-conjugated isotype control antibody (negative). Lineage negative (L-) cells were selected using the Boolean "AND" gate for cells gated as negative for two Confetti markers (P4, P4') and then gated for Sca1 expression as mentioned above. Only RFP+ and GFP/YFP+ cell gating are shown due to space limitation; CFP+ were sorted using a similar gating strategy and selected as GFP/YFP-RFP-CFP+. Note that the YFP and GFP signals are inseparable by FACS.

Supplementary Table 1. Primers used for RT-qPCR analysis

Gene	**Primer**	**Sequence**	**Product size**
Dcn	Forward	TCTTGGGCTGGACCATTTGAA	119
	Reverse	CATCGGTAGGGGCACATAGA	
Lum	Forward	CTCTTGCCTTGGCATTAGTCG	114
	Reverse	GGGGGCAGTTACATTCTGGTG	
Pde1c	Forward	CCTCCTTTGCGTCACTTTAAGC	121
	Reverse	TCATCTTGATCCGCTGATCCA	
Gpc3	Forward	CAGCCCGGACTCAAATGGG	126
	Reverse	CAGCCGTGCTGTTAGTTGGTA	
Hoxa7	Forward	TATGTGAACGCGCTTTTTAGCA	166
	Reverse	GGGGGCTGTTGACATTGTATAA	
3632451O06Rik	Forward	TCATACCAGTATTGGGCTCTC	188
	Reverse	TTCATCCTCTTCATCCTCATC	
Yhwaz	Forward	CGTTGTAGGAGCCCGTAGGTCAT	189
	Reverse	TCTGGTTGCGAAGCATTGGG	
Hprt1	Forward	TGGATACAGGCCAGACTTTGTT	163
	Reverse	CAGATTCAACTTGCGCTCATC	
Hmbs	Forward	ACTGGTGGAGTATGGAGTCTAGATGGC	182
	Reverse	GCCAGGCTGATGCCCAGGTT	
Acta2	Forward	AGATCTGGCACCACTCTTTC	233
	Reverse	GTGAGTCACACCATCTCCAG	
Ly6a/Sca1	Forward	GCTATGGAGTCCCATTTGAG	198
	Reverse	AGGAAGTCTTCACGTTGACC	
