# Supplementary material for: Disease-relevant transcriptional signatures identified in individual smooth muscle cells from healthy mouse vessels
Source: Nat Commun. 2018 Nov 1;9:4567. doi: 10.1038/s41467-018-06891-x (PMC6212435; doi:10.1038/s41467-018-06891-x)
Supplement: Supplementary file 12 — Description of Additional Supplementary Files [file 41467_2018_6891_MOESM12_ESM.docx]

**Title: Supplementary Data 1
Description:** Genes detected as differentially expressed between the aortic arch (AA) and descending thoracic (DT) aorta with bulk RNA-seq

(a) Genes upregulated in AA

(b) Genes upregulated in DT

**Title: Supplementary Data 2. GO term analysis of bulk RNAseq data**

**Description:** (a) GO terms enriched in genes showing preferential expression in the descending thoracic aorta (DT) in the bulk RNA-seq analysis

(b) GO terms enriched in genes showing preferential expression in the aortic arch (AA) in the bulk RNA-seq analysis

**Title: Supplementary Data 3. Random forest classification data**

**Description:** (a) Genes used for final classification

(b) Cells used for classifier training and testing

**Title: Supplementary Data 4. Highly variable genes identified with the variance decomposition-based approach**

**Description:** (a) HVGs detected in AA cells

(b) HVGs detected in DT cells

(c) Functional annotation of the HVGs identified in AA and/or DT based on available literature

**Title: Supplementary Data 5. Top 2% highly variable genes identified with the distance to median approach**

**Description:** (a) Genes identified in AA cells

(b) Genes identified in DT cells

**Title: Supplementary Data 6:
Description:** List of highly variable genes (HVGs) identified in S+L+ cells

**Title: Supplementary Data 7: cVSMC score for cells analysed by Smart-seq2**

**Description:** Cell names reflect cell population: CpSp = S+L+, CnSp = S+L-, CpSn = S-L+

**Title: Supplementary Data 8: List of genes correlating with cVSMC score**

**Description:** (a) Genes that correlate positively with cVSMC score

(b) Genes that correlate negatively with cVSMC score

**Title: Supplementary Data 9: List of GO-terms enriched in genes correlating with cVSMC score**

**Description:** Note: Col B indicates whether the enrichment was found for genes that correlate positively (pos) or negatively (neg) with cVSMC score
